# Based on different immune responses under the glucose metabolizing type of papillary thyroid cancer and the response to anti-PD-1 therapy

**DOI:** 10.3389/fimmu.2022.991656

**Published:** 2022-09-08

**Authors:** Wenjun Xie, Yu Zeng, Linfei Hu, Jiaru Hao, Yuzheng Chen, Xinwei Yun, Qiang Lin, Huashui Li

**Affiliations:** ^1^ Department of General Surgery, Shengli Clinical Medical College, Fujian Provincial Hospital, Fuzhou, China; ^2^ Shengli Clinical Medical College, Fujian Medical University, Fuzhou, China; ^3^ Department of Thyroid and Neck Tumor, Tianjin Medical University Cancer Institute and Hospital, National Clinical Research Center for Cancer, Key Laboratory of Cancer Prevention and Therapy, Tianjin’s Clinical Research Center for Cancer, Tianjin, China; ^4^ Department of Gastrointestinal Cancer Biology, Tianjin Medical University Cancer Institute and Hospital, National Clinical Research Center for Cancer, Key Laboratory of Cancer Immunology and Biotherapy, Tianjin, China; ^5^ Department of Endocrinology, Fujian Provincial Hospital, Shengli Clinical Medical College of Fujian Medical University, Fuzhou, China

**Keywords:** metabolic genes, papillary thyroid cancer classification, immune signatures, prognosis, PD-1, PGBD5

## Abstract

Glucose metabolism-related genes play an important role in the development and immunotherapy of many tumours, but their role in thyroid cancer is ambiguous. To investigate the role of glucose metabolism-related genes in the development of papillary thyroid cancer (PTC) and their correlation with the clinical outcome of PTC, we collected transcriptomic data from 501 PTC patients in the Cancer Genome Atlas (TCGA). We performed nonnegative matrix decomposition clustering of 2752 glucose metabolism-related genes from transcriptome data and classified PTC patients into three subgroups (C1 for high activation of glucose metabolism, C2 for low activation of glucose metabolism and C3 for moderate activation of glucose metabolism) based on the activation of different glucose metabolism-related genes in 10 glucose metabolism-related pathways. We found a positive correlation between the activation level of glucose metabolism and the tumour mutation burden (TMB), neoantigen number, mRNA stemness index (mRNAsi), age, and tumour stage in PTC patients. Next, we constructed a prognostic prediction model for PTC using six glucose metabolism-related genes (PGBD5, TPO, IGFBPL1, TMEM171, SOD3, TDRD9) and constructed a nomogram based on the risk score and clinical parameters of PTC patients. Both the prognostic risk prediction model and nomogram had high stability and accuracy for predicting the progression-free interval (PFI) in PTC patients. Patients were then divided into high-risk and low-risk groups by risk score. The high-risk group was sensitive to paclitaxel and anti-PD-1 treatment, and the low-risk group was sensitive to sorafenib treatment. We found that the high-risk group was enriched in inflammatory response pathways and associated with high level of immune cell infiltration. To verify the accuracy of the prognostic prediction model, we knocked down PGBD5 in PTC cells and found that the proliferation ability of PTC cells was significantly reduced. This suggests that PGBD5 may be a relatively important oncogene in PTC. Our study constructed a prognostic prediction model and classification of PTC by glucose metabolism-related genes, which provides a new perspective on the role of glucose metabolism in the development and immune microenvironment of PTC and in guiding chemotherapy, targeted therapy and immune checkpoint blockade therapy of PTC.

## Introduction

The incidence of thyroid cancer (TC) has been increasing worldwide in recent decades. The most common histologic subtype of TC is papillary thyroid carcinoma (PTC), which is the only histologic subtype of TC that is systematically increasing in all countries (1). The incidence of PTC is almost always higher in women than in men (2). PTC is less malignant than other subtypes of TC, but many patients are still at risk of recurrence and metastasis, at which point the survival rate decreases significantly (3). The construction of prognostic prediction models by abnormally expressed genes as well as nomograms to assess the prognosis of tumour patients and the classification of tumour patients into high-risk and low-risk groups to guide treatment have been developed and applied in several tumourtypes (4, 5). However, reasonable and accurate prognostic prediction models are still lacking in PTC. The current study found that glucose metabolism plays an important role in the development and treatment of PTC (6, 7), and it is unknown whether a reasonable prognostic prediction model can be constructed to predict PTC prognosis and guide PTC treatment by glucose metabolism-related genes in PTC.

Various metabolic pathways have been suggested to play an important role in the development of cancer, such as aerobic glycolysis, glutamine catabolism, and fatty acid metabolism, which produce various nutrients that promote cell growth and proliferation (8–10). Compared to normal tissue, *in vitro* cancer tissue can use large amounts of glucose to produce lactate even in the presence of oxygen, a phenomenon known as aerobic glycolysis or the Warburg effect. Lactate dehydrogenase (LDHA), which is involved in glycolysis, is a transcriptional target of the oncogene MYC and is required for increased glycolysis and tumorigenesis in tumour cells, which provides the molecular basis for the Warburg effect (8). BRAF mutations, which are closely associated with the development of PTC, have also been found to be closely associated with overexpression of several competence metabolism-related genes (11, 12), and inhibition of metabolism-related gene expression has a significant inhibitory effect on PTC progression (13).

The importance of immunotherapy in tumour treatment is constantly being studied, and it forms the basis of cancer treatment together with surgical treatment, radiotherapy and targeted therapy (14). Current studies have found that the metabolism of energy in a variety of tumour cells can affect immune cell function and immunotherapeutic efficacy either by acting directly or influencing the tumour microenvironment (TME). Tumour depletion of glucose metabolically limits T-cell function, leading to their diminished antitumor capacity and thus tumour progression (15). Tumour cells produce large amounts of lactate through aerobic glycolysis and release excess lactate into the TME *via* monocarboxylate transporter protein 4 (16). Lactate in the TME inhibits the therapeutic efficacy of ipilimumab in melanoma (17). Increasing the pH in tumour tissue improves cytotoxic T lymphocyte infiltration and enhances anti-CTLA-4, anti-PD-1 and chimeric antigen receptor (CAR) T-cell therapy (18). However, the effect of glucose metabolism on immune cell infiltration and immunotherapy in PTC has rarely been reported. Therefore, predicting the sensitivity to immunotherapy before administering it to patients with advanced PTC is challenging, but necessary, for individualizing patient treatment and optimizing health care costs.

In this study, we hypothesized that glucose metabolism plays an important role in the development of PTC and modifies the immune microenvironment. Therefore, we clustered information from clinical samples of 501 patients in the TCGA-THCA cohort and 10 glucose metabolism-related pathways. Three subgroups of PTC were identified by unsupervised transcriptome analysis, namely, C1, C2, and C3. Next, we analysed the proportion of PTC subgroups by different clinical stage, mutation type, and frequency of each mutation type. We also compared the tumour mutation burden (TMB), neoantigen number, and mRNA stemness index (mRNAsi) occurring in each of the three subgroups. Then, we further generated the prognostic genes that contributed most to the progression-free interval (PFI) of PTC by Lasso Cox analysis, constructed a prognostic prediction model for PTC based on six prognostic genes (PGBD5, TPO, IGFBPL1, TMEM171, SOD3, TDRD9), constructed a nomogram based on risk score and clinical parameters, divided patients into high risk (HRisk) and low risk (LRisk) groups based on risk score, and then analysed the prognostic differences, immune infiltration, clinical characteristics and sensitivity to drug treatment between the HRisk and LRisk groups. Finally, we evaluated the reliability of the scoring model by observing PGBD5 knock down in PTC. These studies will help to discover the mechanisms of PTC development and guide chemotherapy, targeted therapy and immunotherapy for PTC.

## Materials and methods

### Papillary thyroid cancer patient cohorts

We obtained TCGA-THCA cohort data from the TCGA data portal (https://www.cancer.gov/tcga/), which contains the gene expression profiles of 512 PTC patients. After eliminating the samples with no follow-up time, the gene expression information of 501 patients was finally retained for subsequent analysis. Gene mutation information was also downloaded through the TCGA data portal, and after matching clinical data, gene mutation information was collected for 401 patients. We divided the data of 501 patients into a training set (n=334) and a test set (n=167) based on a 2:1 ratio.

### Gene set variation analysis

We downloaded 10 gene sets related to glucose metabolism from the GO terms and KEGG and REACTOME gene annotation collections from MSigDB (http://www.gsea-msigdb.org/gsea/msigdb/collections.jsp). Then, we used the gene set variation analysis (GSVA) package to calculate the enrichment scores of 10 glucose metabolism-related pathways, used the pheatmap package for clustering analysis, used the limma package in R software for differential analysis of 113 metabolic scores, and defined a signature with an absolute log2-fold change (FC) > 0.2 (adjusted P<0.05) as the differential expression signature. Finally, three subgroups of high, low and medium glucose metabolism activation (C1, C2 and C3) were obtained. Next, we evaluated the different genetic types between the different subgroups, including TMB, neoantigen number, mRNAsi, as well as the mutation types and mutation frequencies in the different subgroups.

### Characterization of PTC subgroups

We identified differentially expressed genes (DEGs) among PTC subgroups using the limma package in R and defined genes with absolute log2FC>1 (adjusted P < 0.01) as DEGs. c2.cp.kegg.v7.2. symbols and h.all.v7.2. symbols downloaded from the Molecular Signature Database (https://www.gsea-msigdb.org/gsea/msigdb) were then used. The symbols gene set files, functional and pathway enrichment analysis was performed using the Cluster Analyser R package, setting the significance threshold to an adjusted P<0.05.

### Screening of prognostic genes and construction of prognostic signatures

The genes associated with PFI of PTC were obtained from TCGA (unicox P < 0.05) and analysed with the DEGs by Venn analysis. Finally, 18 genes associated with the PFI of PTC were obtained. Then, the prognostic genes that contributed most to the PFI of PTC were further generated by Lasso Cox analysis to obtain six glucose metabolism-related genes associated with the prognosis of PTC, and the prognostic profile of glucose metabolism-related genes was constructed using the following formula: risk score = (0.540425503*PGBD5) + (-0.078740238 *TDRD9) + (- 0.281307051 *TMEM171)+(-0.008488446 *IGFBPL1)+(-0.171726033 *TPO)+(-0.001972683*SOD3). Then, the PTC group was divided into the HRisk and LRisk groups according to the expression of six prognostic genes. The prognostic differences, immune infiltration, clinical characteristics and differences in sensitivity to drug treatment were compared between the two groups. And functional and pathway enrichment analysis were performed for both groups by gene set enrichment analysis (GSEA) (19), and GSVA analysis (20).

### Prognostic statistical analysis

The Kaplan-Meier(K-M) analysis was used to calculate the difference in PFI between the HRisk and LRisk groups in the different data sets.The time-dependent receiver operating characteristic (ROC) analysis was used to predict the area under the curve of risk scores for PFI at 1, 3, and 5 years for different data sets. The risk score and clinicopathological features (age, gender, disease stage, and signature) were evaluated by multivariate Cox regression analysis to screen independent risk factors for PFI.

### Construction and evaluation of the nomogram

Using clinical data from all patients, we used the bootstrap self-sampling method to validate the predictive effect of the model using the model itself and then constructed nomogram by the regplot package. We divided the patients into high- and low-scoring groups according to their total points to predict the prognostic differences. The ROC curves, calibration curves, clinical impact curve (CIC) and decision curve (DCA) were applied to evaluate the nomogram’s prediction accuracy and stability.

### Estimation of immune infiltration

The method used to estimate immune infiltration in this study was single-sample GSEA (ssGSEA) and quantified by the R package GSVA using the ssGSEA method. Using a predefined set of genes (usually from functional annotations or results of previous experiments), genes were sorted according to the degree of differential expression in the two types of samples, and then it was tested whether the predefined set of genes was enriched at the top or bottom of this sorting table.

### Prediction of the benefit of each subgroup from chemotherapy, targeted therapy and immune checkpoint blockade therapy

The MD Anderson melanoma cohort that received anti-CTLA-4 or anti-PD-1 therapy was considered for the prediction of immunotherapy response (21). In addition, we performed SubMap (21) analysis of data obtained from the Genomics of Drug Sensitivity in Cancer (GDSC) database (https://www.cancerrxgene.org) to investigate the difference in sensitivity between the HRisk and LRisk groups after treatment with sorafenib or paclitaxel.

### Cell culture and cell transfection

The TPC-1 and KTC-1cell lines were purchased from American Type Culture. All cell lines were identified by short tandem repeat analysis. TPC-1 and KTC-1 cells were cultured in RPMI-1640 medium (Gibco, USA) supplemented with 10% foetal bovine serum (FBS, Biological Industries, Israel), 2 mM L-glutamine (Gibco, USA), penicillin and streptomycin (Gibco, USA). Cells were maintained in a humidified incubator at 37°C and 5% CO2. To study the function of PGBD5, we synthesized a siRNA against PGBD5 (GenePharma, China). The sequence of the synthesized siRNA is shown in **Supplementary Table 1**. TPC-1 and KTC-1 cells were transfected using Lipofectamine 2000 (Invitrogen, USA) according to the manufacturer’s protocol (serum-free medium was used for transfection) and replaced with complete medium containing 10% FBS after 6 h. Cells were harvested for subsequent experiments after continued incubation for 24 h.

### RNA extraction and RT‒qPCR

Total RNA was extracted from cells using TRIzol reagent (Spark Jade, AC0101-B) according to the manufacturer’s instructions. For RT‒qPCR, RNA was reverse transcribed to cDNA by using a Reverse Transcription Kit (Takara, Dalian, China). For RT‒qPCR, PCRs were set up with 2xHQ SYBR qPCR Mix (ZOMANBIO, ZF501) on a 7500 Fast Real-Time PCR System (Applied Biological Systems), and PCRs were performed according to the manufacturer’s description. All samples were normalized to β-actin. The primers used in RT‒qPCR are listed in **Supplementary Table 2**.

### Cell growth and proliferation assays

For cell proliferation assays, an EdU Kit (US EVERBRIGHT, C6015 M) was used according to the manufacturer’s instructions. For the EdU assay, 5×104 cells were plated into 24-well plates and cultured in complete culture medium. After 24 hours, the cells were stained and photographed according to the instructions of the EdU kit.

### Statistical analysis

For normally distributed continuous data, comparisons were made using the t-test, while non-normally distributed data were tested by the Wilcoxon rank sum test. Comparisons between more than two groups of factors were performed using the Kruskal-Wallis test. Differences in survival rates between groups were analyzed by K-M plots and log-rank tests. P values less than 0.05 were considered statistically significant differences. All analyses were performed using R version 4.0.2 (http://www.r-project.org). Statistical analysis of mRNA expression levels and EdU staining levels in PTC cells were performed using the software SPSS 22.0 (SPSS Inc., Armonk, NY, USA). All values are presented as the mean ± standard deviation (SD) of 3 independent replicates. Student’s t test was performed to compare differences. Significant differences were indicated by P<0.05, P<0.01, P<0.001, and P<0.0001.

## Results

### Correlation of PTC subgroups with classical metabolic pathways and clinical features

**Figure 1:**
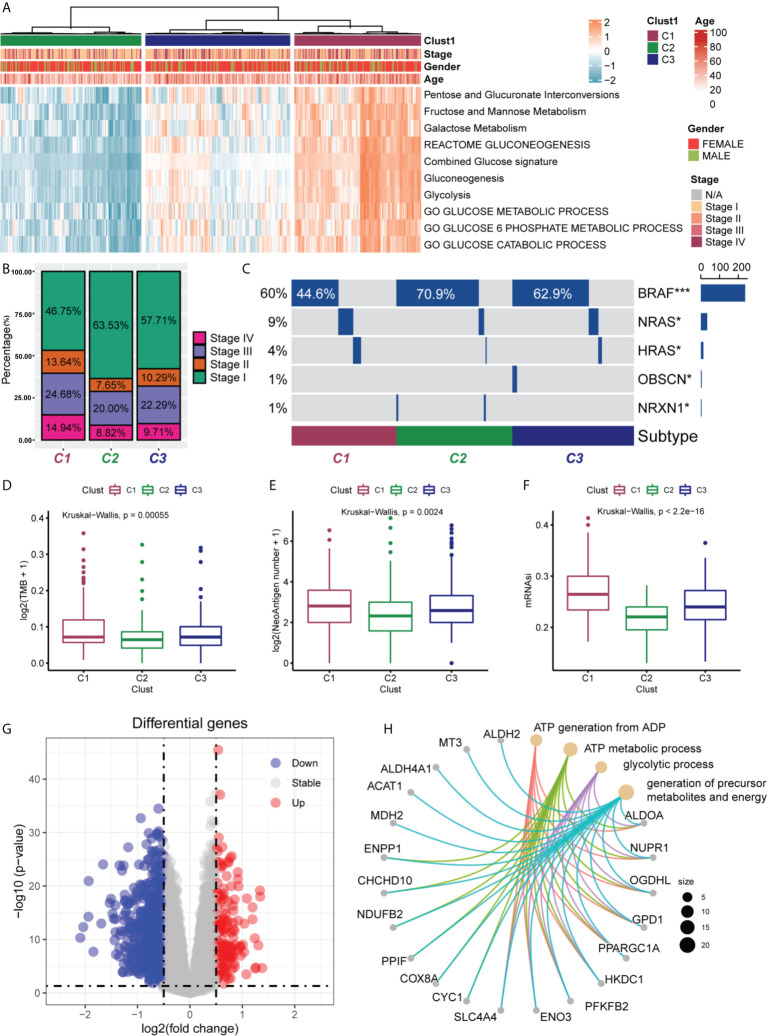
Clinical characteristics of PTC subgroups in TCGA. **(A)** Correlation of the subgroups of PTC patients (C1, C2 and C3) in the TCGA cohort with the 10 glucose metabolism-related pathways and the clinical characteristics of the patients. **(B)** Proportional distribution of the different pathological stages (stage I, II, III and IV) of PTC patients in the three subgroups. **(C)** Oncoprint of the mutational status of the three PTC subgroups. The frequency of NRAS and HRAS mutations were significantly higher in C1 subgroup than in C2 and C3 subgroups. The frequency of classical BRAF mutations was significantly higher in C2 and C3 subgroups than in C1 subgroup. *P < 0.05, ***P < 0.001. Differences in tumour mutation burden (TMB) **(D)**, number of neoantigens **(E)** and mRNA stemness index (mRNAsi) **(F)** among the three PTC subgroups. TMB, neoantigens and mRNAsi were all higher in C1 subgroup than in C2 and C3 subgroups. Statistical differences were compared by the Wilcoxon rank sum test. **(G)** Volcano plot of differentially expressed genes (DEGs) between the C1 and C2 subgroups. A total of 152 DEGs were screened (log2-fold change > 0.5, P < 0.05). **(H)** Enrichment analysis of the 20 most significantly upregulated DEGs.

We first quantified 10 glucose metabolism-related processes using the GSVA R package and then performed differential analysis to find subgroup-specific metabolic profiles. The results of analysis showed that PTC patients could be divided into three subgroups (C1 for high activation, C2 for low activation, and C3 for moderate activation) based on the activation of glucose metabolism-related genes in different metabolic pathways. In addition, the correlation between the different subgroups and the clinicopathological information (age, gender, and clinical stage) of the patients were analysed. In the C1 subgroup and C3 subgroup, the proportion of patients aged >40 was higher, while those aged < 40 were mostly found in the C2 subgroup (**Figure 1A**). This suggested that in patients with PTC, there may be a significant positive correlation between the patient’s age and the level of glucose metabolism in the organism. As shown in **Figure 1B**, the proportions of Stage II, Stage III, and Stage IV were higher in the C1 subgroup than in the C2 and C3 subgroups, suggesting that the higher the level of glucose metabolism activation in their bodies, the higher the patient’s clinicopathological stage. We also observed that NRAS and HRAS mutations were mainly present in the C1 subgroup, while the more classical BRAF mutations occurred mostly in the C2 and C3 subgroups (**Figure 1C**). This illustrated the different responses of these two genomic subtypes when glucose metabolic stresses are different in PTC patients. NRAS and HRAS mutations may be more stimulated in patients when induced with higher glucose metabolic stress, while BRAF mutations are upregulated in the low glucose metabolic activation group.

Next, we also analysed whether there were differences in TMB (**Figure 1D**), tumour neoantigen number (**Figure 1E**), and mRNAsi (**Figure 1F**) in different subgroups. The results showed that TMB, neoantigen number, and mRNAsi were higher in C1 subgroup than in C2 and C3 subgroups, and the differences were statistically significant (all P < 0.05). This suggested that the high glucose metabolic level in PTC patients may be able to promote the production of TMB, neoantigen, and the expression of tumour stemness.

Finally, we performed differential analysis of metabolism-related genes in the C1 and C2 subgroups, setting log2-fold change (FC) >0.5 and P<0.05, and finally screened 152 DEGs (**Figure 1G**). Subsequently, we selected the 20 most significantly upregulated genes (ALDH2, MT3, ALDH4A1. ACAT1, MDH2, ENPP1, CHCHD10, NDUFB2, PPIF, COX8A, CYC1, SLC4A4, ENO3, PFKFB2, HKDC1, PPARGC1A, GPD1, OGDHl, NUPR1 and ALDOA) from 152 DEGs for enrichment analysis and found that these 20 differentially expressed genes were mainly enriched in the generation of ATP from ADP, ATP metabolic processes, glycolytic processes, and the production of precursor metabolites and energy (**Figure 1H**).

### Screening of glucose metabolism-related genes associated with the PFI of PTC

**Figure 2:**
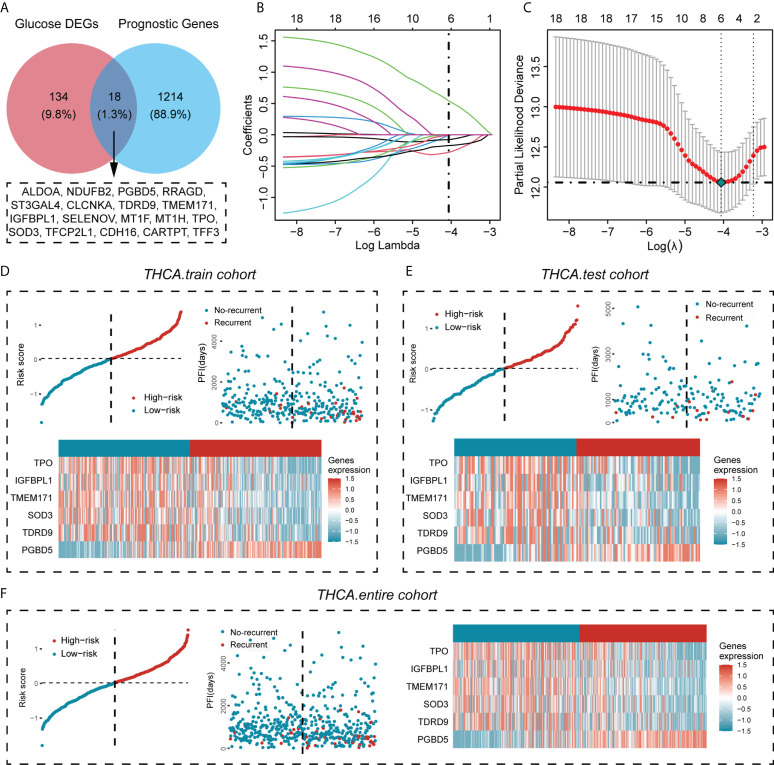
Screening for glucose metabolism-related genes highly associated with PFI and construction of a prognostic prediction model for PTC. **(A)** The genes associated with the PFI of PTC were obtained from the TCGA cohort (unicox P < 0.05) and overlapped with glucose metabolism DEGs, resulting in 18 genes associated with the PFI of PTC. **(B)** LASSO coefficient profiles of the prognostic value. **(C)** Partial likelihood distribution with the corresponding λ-logarithm value. **(D–F)** The distribution of risk scores for the three data cohorts (training cohort, test cohort and entire cohort), the recurrence and nonrecurrence PFIs of the three data cohorts and the heatmap of the expression of six prognostically relevant genes related to glucose metabolism in the High- risk and Low-risk groups.

Next, we obtained the genes associated with PFI of PTC (unicox P < 0.05), and performed Venn analysis with 152 previously obtained glucose metabolism DEGs, and finally obtained 18 genes associated with PFI in glucose metabolism DEGs (ALDOA,NDUFB2,PGBD5,RRAGD,ST3GAL4,CLCNKA, TDRD9,TMEM171,IGFBPL1,SELENOV,MT1F,MT1H,TPO,SOD3,TFCP2L1,CDH16,CARTPT,TFF3) (**Figure 2A**). The prognostic genes that contributed most to the PFI of PTC were then further generated by Lasso Cox analysis, and the formula was constructed as follows: Risk score = (0. 540425503*PGBD5) + (-0.078740238*TDRD9) + (-0.281307051*TMEM171) + (-0.008488446* IGFBPL1) + (-0.171726033*TPO) + (-0.001972683*SOD3) (**Figure 2B, C**). To confirm the strong predictive potential of the prognostic features constructed from the above analysis in different datasets, we used the caret package to divide the TCGA-THCA cohort into training and test sets uniformly and randomly. Next, in the training set, test set and entire set as a whole, we divided the patients into the high risk (HRisk) and low risk (LRisk) groups according to the expression of six glucose metabolism-related genes. We also ranked the patients in each dataset according to the risk score from low to high, and displayed the PFI according to the ranking and the event occurrence of the patients. The PFI of the HRisk group was significantly shorter than that of the LRisk group. Among the six glucose metabolism-related genes screened for prognostic relevance, the expression of five genes (TPO, IGFBPL1, TMEM171, SOD3, TDRD9) was higher in the LRisk group than in the HRisk group, except for PGBD5, which was significantly more highly expressed in the HRisk group than in the LRisk group (**Figure 2D, E, F**).

### Analysis of prognostic differences between different risk groups and the accuracy of ROC curve prediction

**Figure 3:**
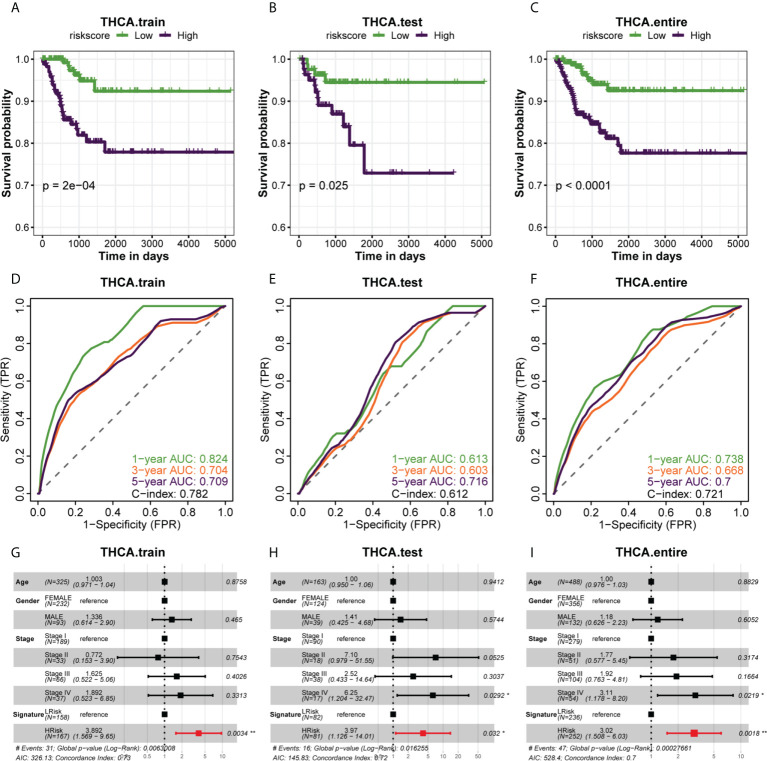
The association of different risk groups with the PFI of PTC. **(A–C)** The Kaplan-Meier survival curve of PFI for the three datasets (training set, test set, and entire set) in the TCGA cohort. Statistical significance of differences was developed by the log-rank test (P < 0.05 for all). **(D–F)** Time-dependent ROC analysis of the three datasets (training set, test set, and entire set) predicted the area under the curve of the risk score for the PFI at 1, 3, and 5 years, respectively. **(G–I)** Multivariate (age, gender, stage, signature) Cox regression analysis of three datasets (training set, test set, entire set).

Since we evenly randomized the PTC cohort into training and test sets by the caret software package, we next divided the patients between the training cohort, test cohort, and entire cohort groups into the HRisk and LRisk groups based on the median risk score of each group and compared the prognosis of PFI between the two groups. The results showed that the prognosis of patients in the HRisk group was worse than that in the LRisk group in all three datasets (all P < 0.05) (**Figures 3A**–**C**), and to further elucidate the accuracy of prognostic features in predicting patients’ PFI, we also performed ROC curve prediction over time. In the training set, the area under the curve (AUC) of the prognostic features reached 0.824, 0.704, and 0.709 at 1, 3, and 5 years, respectively. Similarly, in the test set, the AUC results were 0.613, 0.603, and 0.716 at 1, 3, and 5 years, respectively, and in the entire set, the AUC results were 0.738, 0.668 and 0.700. Overall, our results suggested that the prognostic characteristics of genes related to glucose metabolism can predict the development of PTC with relatively high accuracy (**Figures 3D**–**F**). Finally, a multifactorial Cox regression analysis confirmed the signature of the high risk score as an independent prognostic factor (**Figures 3G**–**I**). The above analysis showed that the risk score constructed by six glucose metabolism-related genes (PGBD5, TPO, IGFBPL1, TMEM171, SOD3, TDRD9) could well predict PFI in PTC patients.

### Construction of the nomogram and evaluation of prediction accuracy

**Figure 4:**
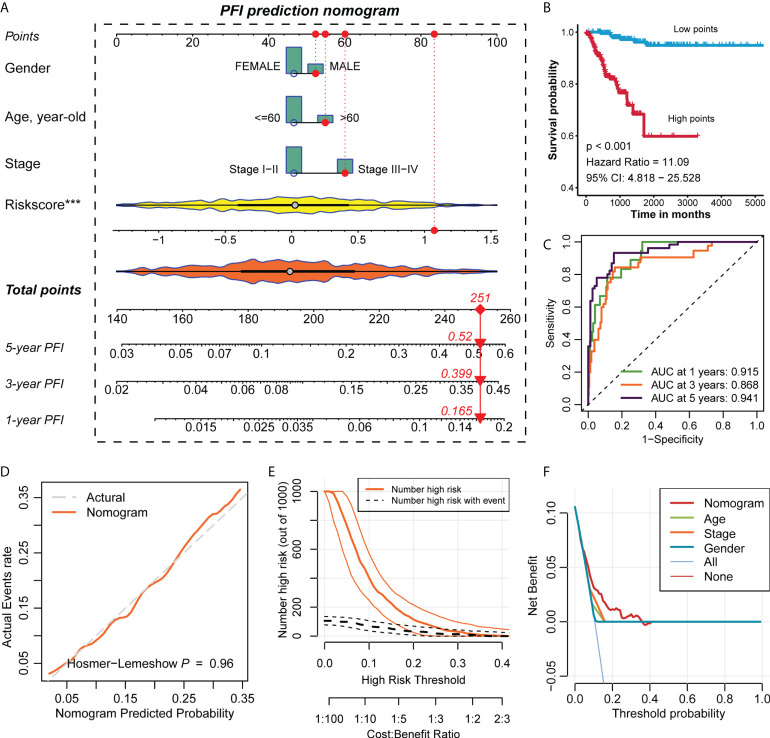
Construction of the nomogram and evaluation of prediction accuracy. **(A)** Construction of the nomogram based on the regplot package for the clinical data of all PTC patients. **(B)** The Kaplan-Meier survival curve of PFI of the high- and low-points groups in PTC. Statistical significance of differences was developed by the log-rank test (P < 0.05). **(C)** Time-dependent ROC analysis predicted the area under the curve of the nomogram for the PFI at 1, 3, and 5 years, respectively. **(D)** The calibration curves of the nomogram. **(E)** The clinical impact curves of the nomogram. **(F)** The decision curve analysis of the nomogram.

We constructed a nomogram based on the regplot package of clinicopathological information of all patients (**Figure 4A**) and classified patients into high- and low-scoring groups according to their total points to compare the prognostic differences and found that patients in the high-scoring group had a worse prognosis than those in the low-scoring group (**Figure 4B**). To further assess the accuracy of the total score in predicting the prognosis of PFI, we also plotted the ROC prediction curves over time. The AUC of the nomogram was 0.915, 0.868 and 0.941 at 1, 3 and 5 years, respectively (**Figure 4C**). Compared with the prognostic prediction model, the AUC of the nomogram was higher at 1, 3 and 5 years, indicating that the nomogram we constructed is highly reliable. The Hosmer-Lemeshow test also demonstrated that the predicted values of nomogram are highly consistent with the true values (p=0.96) (**Figure 4D**). This reflected that the predicted probability of the nomgram is close to the actual probability and the nomogram has an acceptable calibration. In addition, the clinical impact curve (CIC) confirmed that the nomogram accurately predicted the event at risk thresholds from 0-0.3 (**Figure 4E**), and the decision curve analysis (DCA) confirmed that nomogram’s predictive ability was better than clinical indicators (**Figure 4F**).

### Pathway enrichment analysis of HRisk and LRisk and drug sensitivity prediction

**Figure 5:**
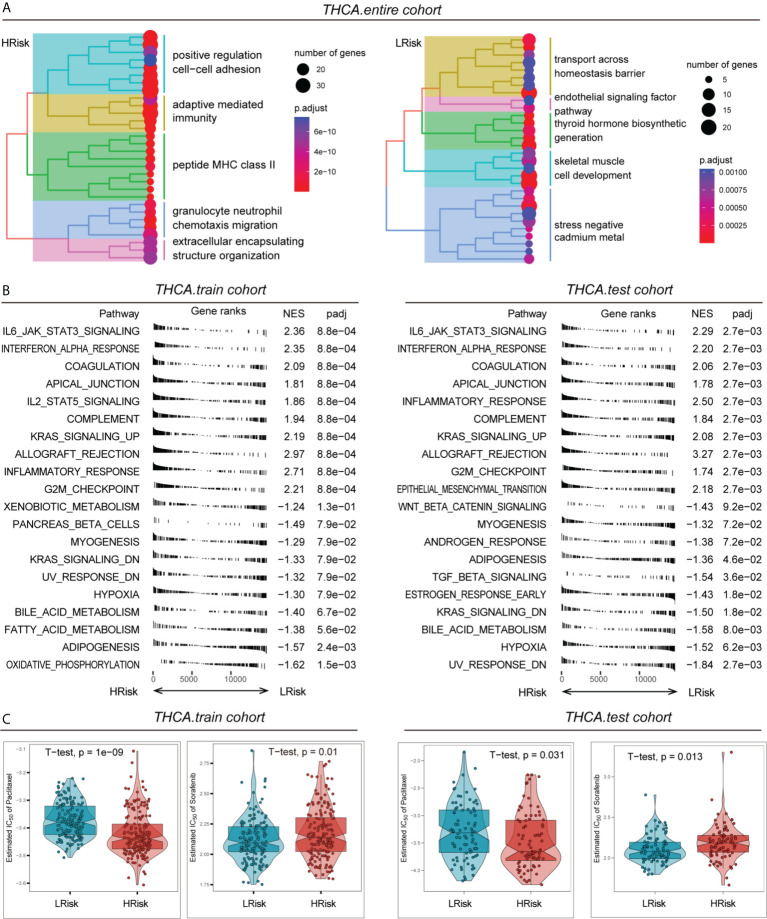
Pathway enrichment and drug sensitivity prediction of different risk groups. **(A)** Differences in the activation of different pathways of the HRisk and LRisk groups in the entire cohort. **(B)** Activation of different HALLMARK pathways in the HRisk and LRisk groups in the training cohort and test cohort. **(C)** Assessment of IC50 values for different drugs (paclitaxel or sorafenib) in the HRisk and LRisk groups in the training cohort and test cohort.

By analysing the differences in enrichment pathways between the overall HRisk and LRisk groups, we found that in the HRisk group, there was activation of different cellular pathways, such as positive regulation of cell‒cell adhesion, adaptive mediated immunity, granulocyte neutrophil chemotaxis migration, and extracellular encapsulating structure organization. In the LRisk group, there was also transport across the homeostasis barrier; in addition, endothelial signalling factor pathway, thyroid hormone biosynthetic generation, and skeletal muscle cell development were also activated in the LRisk group (**Figure 5A**). Then, we further evaluated the activation difference of the HALLMARK pathway between the HRisk and LRisk groups in the training and test set and found that IL6-JAK-STAT3, interferon-alpha response, apical junction and G2M checkpoint were activated in the HRisk group, while myogenesis and hypoxia were activated in the LRisk group (**Figure 5B**). The above enrichment analysis revealed that the level of inflammatory infiltration was higher in the HRisk group. This suggested that PTC patients in the HRisk group may develop metabolic inflammation, and the long-term presence of this inflammation can cause morphological and functional damage to the relevant organs and adversely affect the prognosis of tumour patients.

Paclitaxel is currently used as a first-line chemotherapeutic agent for PTC, while sorafenib is also being used in clinical trials for PTC. Here, we evaluated the response of different risk groups to these two drugs after their use. The results showed that the half maximal inhibitory concentration (IC50) of paclitaxel in the HRisk group was significantly lower than that of the LRisk group, both in the training cohort and the test cohort, while the IC50 of sorafenib in the HRisk group was significantly higher than that of the LRisk group, suggesting that patients in the HRisk group are more suitable for paclitaxel treatment, while patients in the LRisk group may be more suitable for sorafenib treatment (**Figure 5C**).

### Analysis of immune checkpoint differences between HRisk and LRisk

**Figure 6:**
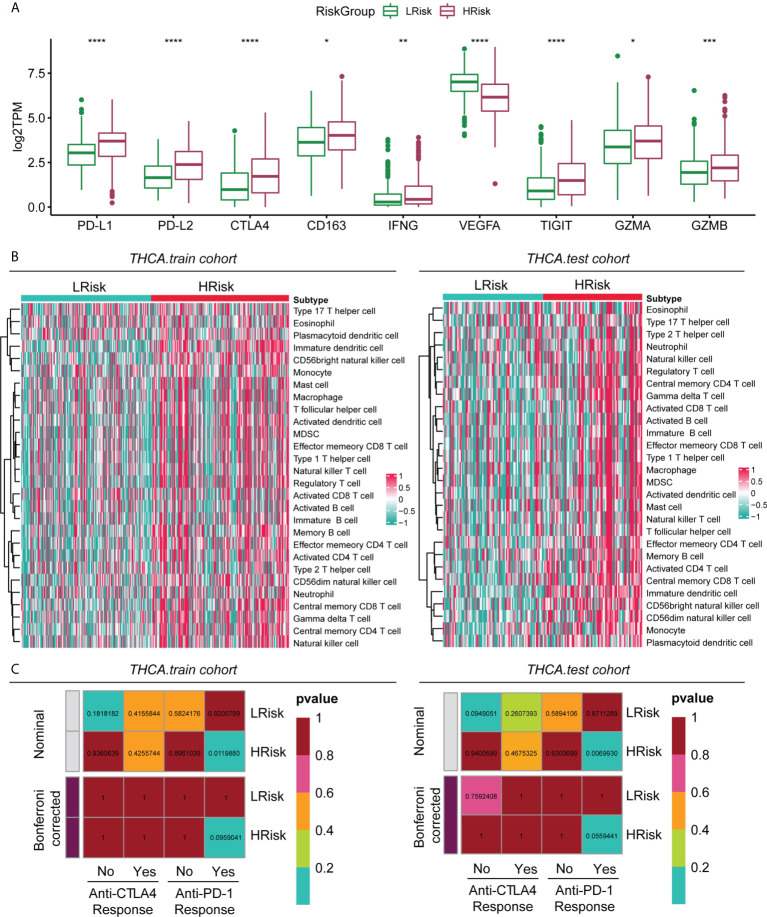
Immune characteristics of different groups in the metadataset. **(A)** Expression level (normalized count) of 9 immune checkpoint genes in the HRisk and LRisk groups. The significant difference was compared through the Kruskal–Wallis test, and the P values are labelled above each boxplot with asterisks (ns represents no significance, *P < 0.05, **P < 0.01, ***P < 0.001, ****P < 0.0001). **(B)** Heatmap describing the abundance of immune and stromal cell populations in the HRisk and LRisk groups. **(C)** Prediction results of the response to anti-CTLA-4 and anti-PD-1 therapy in the HRisk and LRisk groups by subclass mapping analysis.

By analysing the overall immune checkpoint expression differences between the HRisk and LRisk groups, we found that the expression levels of PD-L1, PD-L2, CTLA4, CD163, IFNG, TIGIT, GZMA, and GZMB were all higher in the HRisk group than in the LRisk group, and only VEGFA was higher in the LRisk group (**Figure 6A**). Patients in the HRisk group had tumour cells that expressed a large number of immune checkpoints on their surface. When inhibitory receptors such as PD-L1 and CTLA-4 on the surface of tumour cells are expressed in large numbers, they can deprive T cells of their tumour cell-killing activity, thus enabling immune escape of tumour cells. Enrichment analysis of 28 immune-related gene sets by ssGSEA in the training cohort and test cohort revealed a higher infiltration of immune cells in the HRisk group (**Figure 6B**). The immune checkpoint blockade therapy with anti-PD-1 in the HRisk group of PTC patients may have a better therapeutic effect (P < 0.01) (**Figure 6C**).

### PGBD5 regulates proliferation of papillary thyroid cancer cells

**Figure 7:**
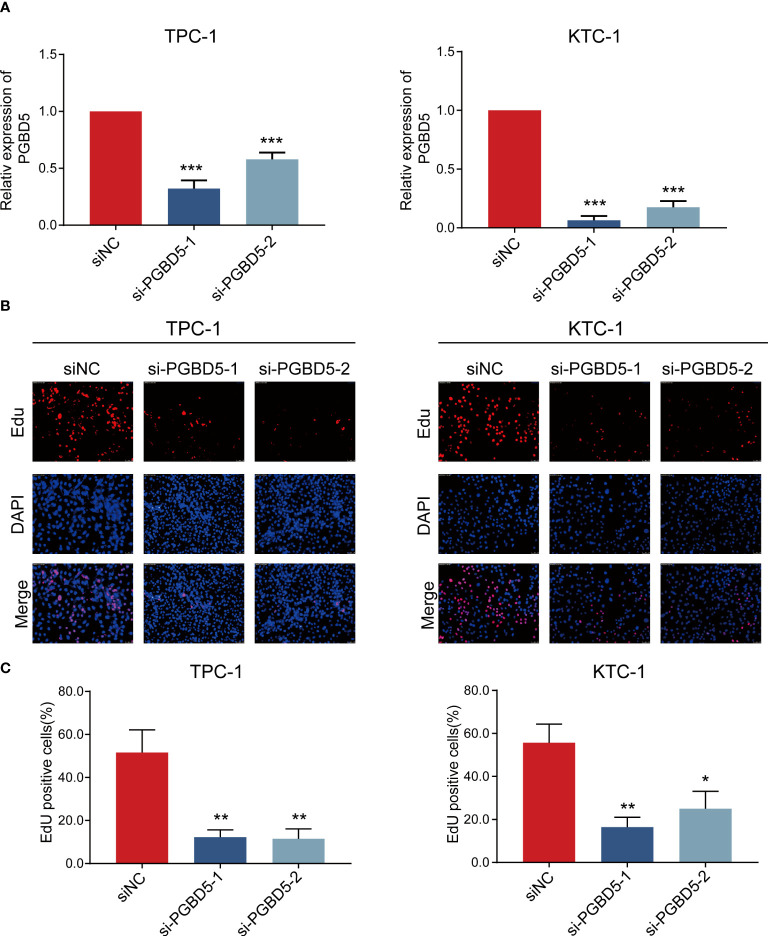
PGBD5 promotes PTC cell proliferation. **(A)** Confirmation of PGBD5 knockdown in TPC-1 and KTC-1 cells by RT-qPCR. **(B)** EdU analysis of TPC-1 and KTC-1cells after the inhibition of PGBD5. *P < 0.05, **P < 0.01, ***P < 0.001.

To verify the accuracy of the above study, we selected PGBD5, which is highly expressed in tumour tissues, for experimental validation. To explore the role of PGBD5 in PTC, we sought to characterize the altered cellular phenotype in PTC cells in the presence of PGBD5 deletion. In both TPC-1 and KTC-1 cell lines, PGBD5 was effectively knocked down by two siRNAs (si-PGBD5-1 and si-PGBD5-2) (**Figure 7A**). The proliferation of PTC cells was significantly reduced after PGBD5 was kncked down, as shown by EdU staining (**Figure 7B**). This demonstrated that PGBD5 was able to promote the proliferation of PTC cells, and when PGBD5 was knocked down, the proliferation ability of the cells was significantly diminished.

## Discussion

PTC is a common malignant tumour. Since PTC is an inert tumour, its prognosis is often better (22). However, most medical treatments are less effective in some patients when distant metastasis occur in PTC (23–25), so accurate prediction of the clinicopathological characteristics and responsiveness to treatments in each PTC patient is the focus of PTC research. Current studies have found that glucose metabolism-related genes play an important role in tumour development and various therapeutic modalities, including immunotherapy (26). To date, no comprehensive analysis has been performed using glucose metabolism-related genes as a prognostic model for PTC. In this study, we first verified that abnormal expression of glucose metabolism-related genes was closely associated with the clinicopathological features of PTC. We then constructed a prognostic prediction model of PTC using glucose metabolism-related genes. Patients were divided into the HRisk and LRisk groups by risk score, and immune cell infiltration and sensitivity to chemotherapy, targeted therapy and immune checkpoint blockade therapy were evaluated in different groups. Meanwhile, we accurately predicted the PFI of PTC patients by prognostic prediction model and nomogram.

Different metabolic pathways are closely associated with the development and prognosis of a variety of tumours (8–10). To determine the clinicopathological characteristics of PTC subgroups that are closely related to glucose metabolic processes, in this study we identified three subgroups of PTC (C1, C2 and C3) by nonnegative matrix decomposition clustering using RNA sequencing data of 2752 genes associated with glucose metabolism. We found that glucose metabolism levels were positively associated with an older age and higher tumour stage in PTC, and both factors predicted a worse prognosis for the C1 subgroup with high glucose metabolism levels. We found that C1 subgroup were associated with higher TMB and higher neoantigens. TMB has a very important prognostic value in immunotherapy as a biomarker of immune checkpoint blockade response (27). Numerous studies have found that high TMB is positively correlated with better treatment outcomes after immunotherapy (28, 29). Neoantigens are abnormal peptides present on the surface of malignant tumour cells that are specifically expressed. Most neoantigens are the products of accumulated mutations in normal cells. Neoantigens have the potential to be recognized by T cells in the context of major histocompatibility complex class (30) and thus exert anticancer effects. Neoantigens have been used in cancer immunotherapy for CAR-T-cell therapy and the design and production of customized vaccines against tumour cells (31). C1 subgroup with higher TMB and more neoantigens may respond better to CAR-T-cell therapy and immune checkpoint inhibitors. C1 subgroup has a higher mRNAsi, which predicts that high glucose metabolism levels are associated with higher levels of PTC stemness, and tumour stemness is closely associated with survival, recurrence, metastasis, and drug resistance in many tumours (32–34). PTC development can be significantly inhibited by suppressing PTC stemness (35), therefore, for the C1 subgroup with high glucose metabolism levels, targeted stemness therapy may achieve good therapeutic results.

Because of the favourable prognosis of PTC, it is difficult to establish a good prognostic prediction model for PTC (36). In this study, we developed a prognostic model containing six glucose metabolism genes (PGBD5, TPO, IGFBPL1, TMEM171, SOD3, TDRD9) to predict the prognosis of PTC. This model has good predictive performance not only in the training cohort but also in the test cohort, indicating the high stability of this prognostic model. This model is an independent prognostic factor for PTC. This model and nomogram performed well in predicting the 1-year, 3-year and 5-year PFIs in PTC patients. Based on the results of the ROC curve analysis, we found that the nomogram outperformed the prognostic prediction model in predicting the PFI of PTC. We verified the reliability of these results by intervening in the transcript levels of glucose metabolism-related genes in PTC in the model.

Among the six glucose metabolism-related genes we screened (PGBD5, TPO, IGFBPL1, TMEM171, SOD3, TDRD9), only PGBD5 had significantly higher expression in the HRisk group. This finding suggests that PGBD5 overexpression may play an important role in the development of PTC. PGBD5 is an active DNA transposase expressed in most paediatric solid tumours and is an important oncogene (37, 38). It has been reported that in rhabdomyosarcoma cells PGBD5 is physically linked to genome-specific signal sequences that promote the induction of DNA rearrangements (39). In addition, a multiomics analysis showed that PGBD5 amplification was associated with poorer overall survival in lobular ductal types of invasive breast cancer (40). We observed a significant inhibition of PTC cell proliferation by siRNA inhibition of PGBD5 expression. This is consistent with the findings of PGBD5 in other tumours. Our present study is the first time to demonstrate that PGBD5 promotes the development of PTC. PGBD5 may be an important target to inhibit the development of PTC in the HRisk group.

The current TNM staging and pathological staging of PTC cannot guide chemotherapy, targeted therapy and immunotherapy for PTC. In this study, PTC was divided into the HRisk and LRisk groups, and we found that the HRisk group was sensitive to paclitaxel and anti-PD-1 therapy. Paclitaxel causes polymerization and stabilization of microtubules mainly by binding microtubule proteins and subsequently inhibits their dynamic properties at the mitotic spindle, which leads to tumour cell block in the G2/M cycle and induces apoptosis (41). We found that genes in the HRisk group are enriched in the G2M checkpoint signalling pathway, which may be the key factor for cell proliferation in the HRisk group. Therefore, paclitaxel may significantly inhibit tumour proliferation by affecting the G2/M phase process in the HRisk group. In the analysis of immune checkpoint gene expression in the HRisk and LRisk groups, we found that PDL-1 was highly expressed in the HRisk group, and that highly expressed PDL-1 could inhibit the migration and proliferation of T cells by binding to PD-1 of T cells, thus inducing tumour tolerance and T-cell failure. Anti-PD-1 could significantly reverse this process to restore the anticancer function of T cells (42); therefore, the HRisk group was more sensitive to anti-PD-1 treatment. The above results suggest that treatment with paclitaxel combined with anti-PD-1 in the HRisk group may lead to better therapeutic outcomes. We found that higher VEGFA expression in the LRisk group and high VEGFA expression were associated with higher lymph node metastasis and more advanced pathological stage in PTC and may promote the transformation of PTC to undifferentiated cancer (43, 44). The VEGF/VEGFR signalling pathway is an important target for the action of sorafenib (45), which could explain the more favourable treatment effect of sorafenib in the LRisk group. Dividing progressive PTC into the HRisk and LRisk groups according to the prognostic prediction model may be advantageous for the selection of appropriate therapeutic agents and more effective individualized treatment.

We must acknowledge the flaws and limitations of our experimental design, which may affect the overall relevance and credibility of our findings. First, as this work was mainly investigated by bioinformatic methods, there may be differences between different algorithms, and more basic and clinical experimental validation is needed to confirm this. Our study suggests that glucose metabolism-related genes are associated with RAS and BRAF mutations, but the mechanisms remain unclear and need to be further explored. In addition, we found that the HRisk group could benefit from paclitaxel and anti-PD-1 treatment, and the LRisk group could benefit from sorafenib treatment, which requires further confirmation of accuracy in clinical trials. Finally, based on our findings, we propose for the first time that PGBD5 could be a therapeutic target to inhibit the progression of PTC, which requires additional studies to further explore its accuracy.

## Conclusion

Our study constructs a PTC prognostic prediction model and proposes a new approach for PTC classification through comprehensive analysis of glucose metabolism-related genes in PTC, providing a new perspective on the role of glucose metabolism in the development and immune microenvironment of PTC and new ideas for guiding chemotherapy, targeted therapy and immune checkpoint blockade therapy in PTC. In addition, we propose for the first time that PGBD5 can be used as a therapeutic target to inhibit PTC progression.

## Data availability statement

The original contributions presented in the study are included in the article/**Supplementary Material**. Further inquiries can be directed to the corresponding authors.

## Author contributions

Conceptualization: WX, HL, QL, LH, JH and YZ. Data curation: WX, JH, QL, and YZ. Formal analysis: WX, HL, YZ, XY, and LH. Funding acquisition: WX. Investigation: WX, XY, QL, and YZ. Methodology: WX, XY, and YZ. Project administration: LH, QL, and HL. Resources: YZ, QL, and JH. Software: WX, YZ, JH, XY, and HL. Supervision: LH and HL. Validation: WX, HL, JH, XY, and YZ. Visualization: HL, JH and WX. Writing – original draft: YC, WX, HL, and JH. Writing – review & editing: all authors. All authors contributed to the article and approved the submitted version.

## Funding

This study was funded by Fujian provincial health technology project (No. 2020QNA007).

## Acknowledgments

Sincerely thank the TCGA database, the Molecular Signature database and the Genomics of Drug Sensitivity in Cancer database and the authors who uploaded the original data. In addition. Thanks to all the authors who contributed to this article, and to the publisher for supporting this article.

## Conflict of interest

The authors declare that the research was conducted in the absence of any commercial or financial relationships that could be construed as a potential conflict of interest.
