# Supplementary material for: Based on different immune responses under the glucose metabolizing type of papillary thyroid cancer and the response to anti-PD-1 therapy
Source: Front Immunol. 2022 Sep 8;13:991656. doi: 10.3389/fimmu.2022.991656 (PMC9536150; doi:10.3389/fimmu.2022.991656)
Supplement: Supplementary file 1 [file Table_1.doc]

**Supplementary Table 1**  The siRNA sequence of PGBD5.

| PGBD5 | sense | anti-sense |
| --- | --- | --- |
| siN.C | UUCUCCGAACGUGUCACGUTT | ACGUGACACGUUCGGAGAATT |
| siPGBD5 1# | GCAGAUACGAUGACAAAUATT | UAUUUGUCAUCGUAUCUGCTT |
| siPGBD5 2# | CAGGCAAGAACUACAUCAUTT | AUGAUGUAGUUCUUGCCUGTT |
