# Supplementary material for: Based on different immune responses under the glucose metabolizing type of papillary thyroid cancer and the response to anti-PD-1 therapy
Source: Front Immunol. 2022 Sep 8;13:991656. doi: 10.3389/fimmu.2022.991656 (PMC9536150; doi:10.3389/fimmu.2022.991656)
Supplement: Supplementary file 2 [file Table_2.doc]

**Supplementary Table 2** The primers of RT-qPCR assays

**PGBD5**

**Forward**5’-GCTTATTCTTCAGCGCATCC-3’

**Reverse** 5’-CAGCCTCTGGGTCAGACAAT-3’

**β-actin**

**Forward** 5′-CTCCATCCTGGCCTCGCTGT-3′

**Reverse** 5′- GCTGTCACCTTCACCGTTCC-3′
